# Enhancing the triage and cohort of patients in public primary care clinics in response to the coronavirus disease 2019 (COVID-19) in Hong Kong: an experience from a hospital cluster

**DOI:** 10.3399/bjgpopen20X101073

**Published:** 2020-04-29

**Authors:** Pang Fai Chan, Kit Ping Loretta Lai, David Vai Kiong Chao, Sau Chun Kitty Fung

**Affiliations:** 1 Consultant, Department of Family Medicine and Primary Health Care, United Christian Hospital, Hong Kong, China; 2 Associate Consultant, Department of Family Medicine and Primary Health Care, Tseung Kwan O Hospital, Hong Kong, China; 3 Consultant and Chief of Service, Department of Family Medicine and Primary Health Care, Kowloon East Cluster, Hospital Authority, Hong Kong, China; 4 Consultant, Department of Pathology, United Christian Hospital, Hong Kong, China

**Keywords:** public primary care clinics, COVID-19, triage and cohort, primary health care

## Introduction

The current COVID-19 outbreak has been characterised by the World Health Organisation (WHO) as a pandemic, as of 11 March 2020.^1^ The rapid spread and severity of the disease represents a global threat. Despite being geographically very close to the mainland China, where there were more than 80 000 confirmed cases, Hong Kong had successfully contained the number of confirmed cases to the relatively small number of 936 as of 7 April 2020. Among the confirmed cases, 538 (57.5%) were imported cases, 163 (17.4%) were local or possibly local cases, and 235 (25.1%) were close contacts of confirmed cases. Around 6% of patients had serious disease requiring intensive care, and four patients (0.4%) have died from the disease.^2^ Success in controlling the outbreak so far is likely due to proactively implemented government policies on travel and border controls; school and temporary work suspension; high level of public vigilance including the practice of wearing face mask in public areas;^3^ and good infection control in healthcare settings.^4^ In primary care settings, the prompt detection and effective triage and cohort of potentially infected patients are essential to prevent nosocomial infections among patients, healthcare workers, and visitors.^5^

## Method

### The risks of COVID-19 in public primary care clinics in Hong Kong

In Hong Kong, there are 73 General Outpatient Clinics (GOPCs) under the management of the Hospital Authority (HA), providing subsidised primary care services for patients with both episodic illnesses and chronic diseases. All healthcare workers in GOPCs received regular training and audit on infection control, including the proper use of personal protective equipment (PPE). During emergency response level for epidemic or pandemic of serious infectious disease, enhanced measures for infection prevention and control would be implemented, including: reinforcement of standard, contact, droplet, and airborne precautions (when performing aerosol generating procedures) for patient care; universal masking; provision of alcohol-based hand rub at convenient locations; posting of educational posters; and regular public announcement for infection prevention. The frequency of environmental cleaning, decontamination, and equipment disinfection in high-risk areas would also be enhanced.

In GOPCs, most medical appointments were prescheduled after previous visits for chronic illnesses or booked by an interactive telephone appointment system within 24 hours for episodic illnesses, including a lot of acute respiratory illnesses. Therefore, clinics would not know before patient arrival whether they were having symptoms that might be related to COVID-19. In each GOPC, there was a fever consultation room equipped with a special ventilation requirement of at least six air changes per hour and negative airflow from waiting area to the fever consultation room. High-efficiency particulate air (HEPA) filters were also installed in some fever consultation rooms. In usual operation, patients presenting with fever and influenza-like symptoms together with epidemiological criteria of avian influenza, Middle East respiratory syndrome (MERS), or other highly infectious airborne respiratory disease would be triaged, cohorted, and then assessed by a doctor wearing necessary PPE in the fever consultation room. However, studies showed that a large proportion of patients infected with COVID-19 would only have mild symptoms without fever before hospitalisation.^6–8^ By using the original triage arrangement, some infected but afebrile patients would not be triaged out and cohorted. Allowing the infected patients to stay together with other visitors in a busy and crowded clinic waiting area would put the other people at risk, especially older patients with chronic conditions.

Moreover, in view of the global shortage of PPE supply, it was recommended by the WHO^9^ and Hospital Authority of Hong Kong (Table 1) that PPE should be used judiciously and according to different risk levels. On the other hand, studies showed that healthcare associated infections were not uncommon in both hospital and outpatient clinics.^10,11^ In facing a potential life-threatening infectious threat, healthcare workers would have a lot of anxiety.^12^ The authors observed that some healthcare workers, including doctors, were wearing escalated levels of PPE at ’non-high risk’ areas, as they worried about infection after contact with hidden patients with mild symptoms and their infective droplets. The risk of infection to doctors would also be higher due to frequent and hurried donning and doffing when seeing patients in and out of fever consultation room, and this resulted in inconsistent removal procedure of PPE.^13^ Therefore, early detection and cohort of patients suspected to have infections with the novel coronavirus were important to protect the healthcare workers so that they could manage the suspected cases with appropriate PPE in clinic areas equipped with ventilation system fulfilling the infection control requirement.^14,15^

**Table 1.** Recommended PPE in different settings by Hospital Authority in Hong Kong (updated on 18 Feb 2020)

	AIIR for suspected/ confirmed COVID-19	Triage station / fever consultation room at GOPC	Surveillance ward/ cubicle/ side-room	Aerosol- generating procedures (AGPs)	Other patient areas(eg, other wards or general consultation room)	Other area with no direct patient contact
Hand hygiene	Yes	Yes	Yes	Yes	Yes	Yes
Surgical mask	N95	Surgical mask/ N95	Surgical mask/ N95	N95	Yes	Yes
N95 respirator					Standard precautions ±transmission based precautions	No
Isolation gown	AAMI level 1	AAMI level 1	AAMI level 1	AAMI level 1		No
Disposable gloves	Yes	Risk assessment	Risk assessment	Yes		No
Eye protection	Goggles/ face shield	Eye visor/ goggles/ face shield	Eye visor/ goggles/ face shield	Goggles/ face shield		No
Hair cover	Optional	Optional	Optional	Optional		No

AMMI level 3 isolation gown can be considered when splashing is anticipated. Alternatively, a waterproof apron on top of the AMMI level 1 isolation gown is also acceptable. Shoe covers are not recommended.

AAMI = The Association for the Advancement of Medical Instrumentation. AIIR = airborne infection isolation room. PPE = personal protective equipment. GOPC = General Outpatient Clinics.

In order to reduce the risk of nosocomial infections among healthcare workers and other patients, an enhanced triage and cohort system was implemented in one of the seven clusters of Hospital Authority in Hong Kong from 25 February 2020.

### Enhanced triage and cohort system in GOPCs

There were eight GOPCs in the cluster providing around 18 000 consultations per week. Each clinic had designed a specific patient route for patient cohort and admission arrangement. The detail operation logistics are summarised below.

#### Triage

Clinics would close all side entrance so that all visitors must enter the clinics through the main entrance. All visitors must wear a surgical mask and disinfect both hands with alcohol-based hand rub before entering the clinics. Body temperature would be measured by infrared forehead thermometer at the triage station and rechecked with a tympanic thermometer if screening was positive. Patients would then be triaged by asking them for any fever, acute respiratory symptoms, and travel history in the past 2 weeks. Patients with acute respiratory symptoms and/or fever would be cohorted in a special waiting area, separate from the waiting area for other patients. Patients with travel history to areas with community outbreak of COVID-19 and feeling unwell would also be cohorted.

#### Cohort

Each GOPC would set up a well-ventilated cohort waiting area (Special Waiting Area) for cohorting patients with acute respiratory symptoms and/or fever, in alignment with the WHO, UK, and US recommendations.^16–18^ Patients would be escorted by healthcare workers to the Special Waiting Area and any concerns about the arrangement raised by patients would be addressed by detailed explanations. Patients must keep wearing their surgical masks and would be arranged to sit with appropriate separation distance (that is, at least 1 metre according to WHO recommendation) whenever possible.^19^ A surgical mask would be provided to patients wearing a non-medical mask which might not provide adequate protection against droplets or to contain respiratory secretions (for example, wearing a reusable mask made of cotton cloth or a mask with obvious contamination). Alcohol-based hand rub for hand disinfection and a waste receptacle for disposing used tissue paper were provided inside the Special Waiting Area.

All patients would be further assessed by a nurse wearing appropriate PPE according to the latest Fever-Travel-Occupation-Contact-Clustering (FTOCC) triage checklist for novel serious respiratory infections (Supplementary Appendix 1). Patients requiring nursing assessment such as blood pressure measurement or haemoglucotest would be assessed by the same triage nurse in the Special Waiting Area. The triage nurse would then inform the fever consultation room doctor about consultation, with urgent arrangement for patients who had fever or history fulfilled the reporting criteria by FTOCC checklist.

#### Consultation

Doctors in general consultation rooms would perform the second triage by asking for the presence of fever, acute respiratory symptoms, and travel history at the beginning of each consultation, to capture cases missed at the triage station. A travel history indicator in the computerised medical record system would turn red if patients had any travel history in the past 14 days. Any screened positive patients would be escorted by the triage nurse to the Special Waiting Area for detailed FTOCC triage and subsequent doctor consultation in the fever consultation room.

Each clinic would arrange one designated doctor in each session to see all cohorted patients in the fever consultation room with recommended PPE. The doctor was not required to perform doffing before seeing next patient unless the PPE was contaminated or after attending a case fulfilling the reporting and hospital admission criteria. If there was any suspected case requiring hospital admission, the patient would be isolated in a designated consultation room or waiting area with good ventilation while waiting for ambulance transportation. After admission to hospital, nasopharyngeal and throat swab would be taken by a nurse in an airborne isolation room for reverse transcription polymerase chain reaction (RT-PCR) test for SARS-CoV-2. For patients not indicated for admission, symptomatic treatment and health advice including good personal and environmental hygiene for respiratory tract infection would be provided. Patients would also be advised to seek medical consultation if they had persistent or deteriorating symptoms.

After consultation for suspected cases, cases with body fluid spill, and after each consultation session, the consultation room would be cleaned and decontaminated according to the prevailing infection control guidelines.

The patient flow for triage and cohort is illustrated in the flowchart in Supplementary Appendix 2.

## Results and discussion

Out of 76 934 patients attending the eight GOPCs for consultation during the first 6 weeks of implementation of the new system (from 25 February 2020 to 6 April 2020), 6 539 patients (8.5%) were triaged and cohorted and seen in the fever consultation room. Among the cohorted patients, only 350 (5.4%) patients had fever. Seventy-nine patients (1.2%) fulfilled the reporting criteria for COVID-19 and were admitted to hospital for molecular testing; 128 patients (2.0%) with clinical diagnosis of pneumonia were referred to the Accident and Emergency department for further radiological investigations, and none of them were diagnosed with COVID-19. Five patients who had attended GOPCs were confirmed to have COVID-19 infection.

There was no negative feedback about the enhanced cohort arrangement received from patients. With the implementation of the enhanced triage and cohort system in all eight GOPCs, the authors observed that all healthcare workers followed the PPE recommendations well as they were reassured and felt better protected under the enhanced system. No staff was required to be quarantined due to the absence of appropriate PPE during close contact with the confirmed cases, and there was no reported nosocomial infection.

A large amount of PPE was saved for use in other high risk areas or procedures in hospitals, with the weekly consumption of PPE significantly dropping after the implementation of the new system (Table 2).

**Table 2.** Comparison of weekly consumption of PPE in 8 General Outpatient Clinics before and after the implementation of the new system on 25 February 2020

Period	PPE used
N95respirators	Isolation gown	Face shield
3–8 Feb 2020	1546	1736	1377
9–14 Mar 2020	184	815	256
Percentage drop in usage	88.1%	53.1%	81.4%

PPE = personal protective equipment.

Since most (82.6%) confirmed cases were either imported cases or with close contact history with confirmed cases in Hong Kong, a robust triage system including the use of a comprehensive triage checklist was effective in screening out high risk patients for further sensitive diagnostic tests without overloading the hospitals. However, in the event of a larger number of confirmed cases found to have no travel or contact history, providing a sensitive molecular test to those patients presenting with mild symptoms of COVID-19 in outpatient settings should be considered.

## Conclusion

With the announcement of COVID-19 pandemic by WHO, healthcare professionals are facing the threat of encountering more patients infected with the disease in public primary care clinics. A robust triage and cohort system is of utmost importance to help early detection of suspected cases and reduce the risk of community outbreak in the containment phase. The enhanced system could also reduce the risk of nosocomial infections and to ensure effective use of PPE in primary care.
